# Metabolic Remodeling in Glioma Immune Microenvironment: Intercellular Interactions Distinct From Peripheral Tumors

**DOI:** 10.3389/fcell.2021.693215

**Published:** 2021-06-11

**Authors:** Runze Qiu, Yue Zhong, Qingquan Li, Yingbin Li, Hongwei Fan

**Affiliations:** ^1^Department of Clinical Pharmacology Lab, Nanjing First Hospital, Nanjing Medical University, Nanjing, China; ^2^Center of Drug Discovery, State Key Laboratory of Natural Medicines, China Pharmaceutical University, Nanjing, China; ^3^Department of Neurosurgery, The Second Affiliated Hospital of Nanjing Medical University, Nanjing, China

**Keywords:** glioma, metabolic reprogramming, tumor microenvironment, immune escape, metabolic therapy

## Abstract

During metabolic reprogramming, glioma cells and their initiating cells efficiently utilized carbohydrates, lipids and amino acids in the hypoxic lesions, which not only ensured sufficient energy for rapid growth and improved the migration to normal brain tissues, but also altered the role of immune cells in tumor microenvironment. Glioma cells secreted interferential metabolites or depriving nutrients to injure the tumor recognition, phagocytosis and lysis of glioma-associated microglia/macrophages (GAMs), cytotoxic T lymphocytes, natural killer cells and dendritic cells, promoted the expansion and infiltration of immunosuppressive regulatory T cells and myeloid-derived suppressor cells, and conferred immune silencing phenotypes on GAMs and dendritic cells. The overexpressed metabolic enzymes also increased the secretion of chemokines to attract neutrophils, regulatory T cells, GAMs, and dendritic cells, while weakening the recruitment of cytotoxic T lymphocytes and natural killer cells, which activated anti-inflammatory and tolerant mechanisms and hindered anti-tumor responses. Therefore, brain-targeted metabolic therapy may improve glioma immunity. This review will clarify the metabolic properties of glioma cells and their interactions with tumor microenvironment immunity, and discuss the application strategies of metabolic therapy in glioma immune silence and escape.

## Introduction

Glioma is the most common primary intracranial cancer with a 5-year survival rate of less than 10% (Wang J. et al., 2019), occurring in glial cells such as astrocytes, oligodendrocytes and microglia. Glioblastoma multiforme (GBM) arose from astrocytes is the most frequent glioma with high malignancy and drug resistance, which was classified as grade IV in the WHO grade 2016, with a 5-year relative survival rate of only 5% because of rapid relapse after treatment (Gusyatiner and Hegi, 2018). According to gene transcription characteristics, GBM can be further classified into three subtypes: proneural [mutations on isocitrate dehydrogenase (IDH)-1 or tumor suppressor p53, and PDGFRA amplification], mesenchymal (mutation/deficiency of tumor suppressor NF1), and classical [EGFR amplification and CDKN2A (Ink4a/ARF) homozygous deletion] (Wang Q. et al., 2017). NF1 mutation-mediated proneural-mesenchymal transition is the key mechanism of relapse, causing resistance to treatment (Behnan et al., 2019).

Operative resection, although improving overall survival and prognosis in patients with low- and intermediate-grade gliomas (LGGs and IGGs) (Hervey-Jumper and Berger, 2016), shows limited effect on high-grade gliomas (HGGs) including anaplastic gliomas (WHO grade III) and GBM. Ironically, due to the changeable biological properties and the location of gliomas, non-specific interventions including radiation and brain-permeable cytotoxic drugs benefited patients even more than targeted therapies (Chen R. et al., 2017; Touat et al., 2017). Nevertheless, the tumor microenvironment (TME) makes gliomas resistant to chemotherapeutic drugs, and bring about inflammation to further reduce the prognosis of patients (Wu and Dai, 2017; Yang and Lin, 2017). In order to satisfactorily treat gliomas, one needs to be familiar with brain TME, which determine the evolution of tumors (Hirata and Sahai, 2017).

**FIGURE 1:**
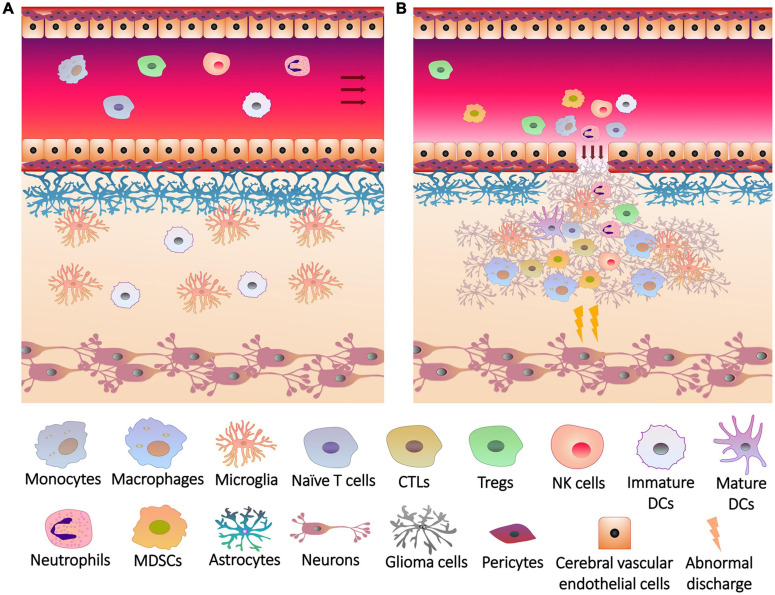
The breakage of blood brain barrier and the infiltration of immune cells into glioma. **(A)** Under physiological conditions, the blood brain barrier consists of a firm multilayer barrier. Cerebral vascular endothelial cells form a tight junction structure, which is closely connected with pericytes, and is supported by basement membrane underneath. Astrocytes wrap the basement membrane through the end foot, and microglia maintain the integrity of the barrier. The immune cells originally present in the brain are mainly microglia and dendritic cells (DCs). Peripheral immune cells cannot penetrate the blood brain barrier, and brain tissues do not release recruitment signals to the cerebral blood vessels. **(B)** Rapid growth of glioma cells not only overexcites neurons to induce seizures, but also injures blood brain barrier. Glioma cells release chemokines and other cytokines through the cracks of blood brain barrier to induce the differentiation, expansion and recruitment of peripheral immune cells, including monocytes (in blood)/macrophages (differentiated from monocytes in brain tissue), cytotoxic T lymphocytes (CTLs), regulatory T cells (Tregs), natural killer (NK) cells, neutrophils, DCs and myeloid-derived suppressor cells (MDSCs), which infiltrate the lesions with immune cells *in situ*.

Since peripheral immune cells cannot enter the blood-brain barrier (BBB) and release inflammatory factors into brain under physiological conditions, brain tissues are protected from inflammation (Engelhardt et al., 2017; Figure 1A). Glial cells play an important role in the integrity and damage repair of BBB (Lou et al., 2016), so glioma cells originating from glial cells can impair BBB and allow peripheral immune cells to enter the brain (Figure 1B), forming a unique TME together with intracranial situ cells, tumor-associated vasculature, perivascular niche and lymphatic vessels (Quail and Joyce, 2017). Immune cells are major members of the glioma TME (Magana-Maldonado et al., 2016; Gieryng et al., 2017), which gradually lost tumor clearance duties or became associates when exposed to tumors. To support tumor growth, glioma cells and glioma stem-like cells (GSCs, also known as brain tumor initiating cells) interacted with adaptive immune cells and recruited immunotolerant innate immune cells to inhibit or evade anti-tumor responses (Broekman et al., 2018).

Although the BBB was damaged, glioma TME reconstructed the blood-brain tumor barrier (BBTB), whose structure is still dense and makes it difficult for antibodies to enter the lesion (van Tellingen et al., 2015). This may be the reason why immunotherapy such as PD-1/PD-L1 monoclonal antibody was less effective in glioma (Jackson et al., 2019). In contrast, metabolic therapies, such as ketogenic diet, significantly benefited patients (Winter et al., 2017). Metabolism is a medium of the communications between glioma and immune cells in TME (Thomas and Yu, 2017). Glioma cells interfere with immune cells through heterogeneous metabolism to mediate tumor growth, invasion, drug resistance and recurrence, which will be reviewed below.

## Metabolic Properties of Glioma Cells

Compared with normal tissues, tumors have specific anabolic and catabolic needs due to their rapid and uncontrolled growth. After metabolic reprogramming, tumors tend to gain energy through glycolysis rather than oxidative phosphorylation (OXPHOS) even under aerobic conditions (DeBerardinis and Chandel, 2016), which is called Warburg effect. Recent studies have found that glioma cells that initially grew in an ischemic environment relied on aerobic pentose phosphate pathway (PPP) instead of glycolysis after being exposed to adequate oxygen (Kathagen-Buhmann et al., 2016). When glucose was depleted, glioma cells re-converted the metabolic pattern to OXPHOS via the accumulation of lactate, which is the by-product of glycolysis (Duan et al., 2018). In addition to differentiated glioma cells, GSCs can also switch metabolism between glycolysis and OXPHOS catering to changing circumstances (Shibao et al., 2018), suggesting that the metabolism of glioma cells is environmentally plastic.

Mutations or changed levels of metabolic enzymes and accumulation of metabolites in TME were associated with the malignant progression and epigenetic modifications (Agnihotri and Zadeh, 2016; Masui et al., 2019). Isocitrate dehydrogenase (IDH), a key rate-limiting enzyme in tricarboxylic acid (TCA) cycle regulating carbohydrate, lipid and amino acid metabolism, was widely mutated in proneural GBM cells. Mutations in gliomas often occur in IDH1 and IDH2, both of which caused the conversion of α-ketoglutarate (α-KG) to 2-hydroxyglutarate (2-HG) in a NADPH-dependent manner (Waitkus et al., 2016). The overall survival of patients with proneural subtypes is longer than other subtypes, but the survival became the shortest after excluding IDH mutations, suggesting the positive significance of IDH mutations for prognosis (Behnan et al., 2019). However, IDH mutations may lead to the conversion of LGG to secondary HGG and the emergence of hypermutation phenotypes (Han et al., 2020). As another feature of proneural subtypes, mutations in p53 led to the loss of glycolysis inhibition and promoted tumor cells to adapt to hypoxic environments (Maurer et al., 2019). The expansion of PDGFRA also promoted glycolysis of proneural subtypes (Ran et al., 2013). In the classical GBM subtype, overexpression of EGFR activated PFK1 through PI3K-AKT signaling, leading to the upregulation of GLUT1 and the enhancement of glucose uptake (Lee et al., 2018). Although there is no direct evidence that the inactivation of tumor suppressor NF1 is associated with enhanced glycolysis, the glucose uptake and glycolysis of mesenchymal GBM cells are more active than other subtypes, which explains the increase in the malignancy of GBM caused by proneural-mesenchymal transition. The accumulation of ROS caused by hypoxic lesions can induce this transition, and PI3K-AKT was also stimulated under hypoxia, resulting in active glucose uptake and glycolysis (Talasila et al., 2017; Liu et al., 2020).

Dietary or pharmacological interventions on metabolism, such as ketogenic diet (Poff et al., 2019), dimethylbiguanide, statins and NSAIDs (Gerthofer et al., 2018; Seliger and Hau, 2018) inhibited the growth and invasion of HGGs and the malignant transformation of LGGs, and induced programmed tumor death. In view of the role of cell metabolism in the progression of gliomas, means of metabolomics based on high-throughput analysis has been developed currently (Pandey et al., 2017), whose application requires the familiarity with the metabolic characteristics of glioma cells and GSCs.

### Carbohydrate Metabolism

The metabolic trend of glioma cells switches between glycolysis and PPP according to the concentration of oxygen. Under hypoxia, glioma cells overexpressed glycolytic enzymes to maintain energy supply and promote migration, while up-regulating PPP enzymes for rapid proliferation and division under oxygen-rich condition (Kathagen-Buhmann et al., 2016; Kathagen-Buhmann et al., 2018). Glycolysis was highly activated in HGG cells with invasiveness and resistance to conventional treatment (Corbin et al., 2017), but weaker glycolysis was detected in LGG cells with IDH1 mutations, which restricted their energy and made them less aggressive (Fack et al., 2017). Intracellular glucose was catalyzed to glucose-6-phosphate (G6P) by hexokinase (HK), followed by the transformation to 2 glyceraldehyde 3-phosphate (GA3P) via glucose-6-phosphate isomerase, 6-phosphofructokinase (PFK)-1, aldolase and triose phosphate isomerase sequentially, with the cost of 2 ATP. Then GA3P was converted to pyruvate catalyzed by glyceraldehyde 3-phosphate dehydrogenase, phosphaglycerate kinase (PGK), phosphoglycerate mutase 1, enolase and pyruvate kinase M (PKM), during which 4 ATP were generated. The product pyruvate was aerobically converted to acetyl-CoA to enter TCA cycle via pyruvate dehydrogenase (PDH), or became lactate by lactic dehydrogenase (LDH)-A without oxygen. The level of lactate was positively related to the speed of glycolysis, which was significantly higher in HGG cells than neuroblastoma cells and neurons (Kim J. et al., 2015). Apart from energy supply, abundant glycolytic enzymes in malignant glioma cells and GSCs promoted the shift of carbon to ribose-5-phosphate (R5P) for the synthesis of nucleotide (Agnihotri and Zadeh, 2016), supporting tumor growth. To ensure enough raw materials for glycolysis, glioma cells and GSCs took up glucose efficiently via elevated glucose transporter (GLUT) (Libby et al., 2018), of which the level of GLUT3 is characteristic (Xu et al., 2015; Zheng et al., 2016), relating to the resistance to antiangiogenic drugs (Kuang et al., 2017). Glucose is not the only carbohydrate source for glioma cells. They also ingested fructose through GLUT5 and utilized by ketohexokinase for limited growth (Gao et al., 2018; Su et al., 2018).

Carbohydrate uptake and glycolysis of glioma cells were driven by the PI3K–AKT pathway activated by receptor tyrosine kinases including EGFR and c-Met, which up-regulated PFK1 and GLUT1/3 via the activation of PFK2 and glycoprotein synthase kinase (GSK)-3β (Kuang et al., 2017; Lee J.H. et al., 2017; Lee et al., 2018). The mTOR–c-Myc signaling also facilitated glycolysis by glutamine–fructose-6-phosphate aminotransferase 1 (Liu B. et al., 2019). In contrast, the AMP-activated protein kinase (AMPK) inhibited glycolysis and glucose uptake by inhibiting mTORC1 and attenuating transcription coactivator yes-associated protein-induced GLUT3 expression (Wang et al., 2015). As a downstream effector of mTORC2 and a regulator of the PI3K–AKT signaling (Oh et al., 2017; Wang G. et al., 2017), hypoxia-inducible factor (HIF)-1α induced the production of GLUT1/3 and glycolytic enzymes such as HK2 and PDH kinase 1 to drive glucose uptake and glycolysis, and reduced reactive oxygen species (ROS) to resist oxidative stress (Yang et al., 2014; Gabriely et al., 2017). Augmented levels of microRNAs that activate PI3K–AKT–mTOR and mTORC2–c-Myc axis and repress AMPK signaling have been observed in malignant glioma cells (Alfardus et al., 2017), reflecting the transcriptional activation of glycolysis and glucose transport.

As an important part of anabolism, PPP was activated by receptor tyrosine kinases–mTOR pathway by phosphorylated 6-phosphogluconate dehydrogenase (Liu R. et al., 2019), using G6P to produce substrates needed for glioma growth. The consumption of each G6P via 6-phosphogluconate dehydrogenase and 6-phosphate gluconate dehydrogenase produced 1 CO2, 3 H^+^ and 2 NADPH, maintaining tumor growth by adjusting pH and producing NAPDH for the synthesis of GSH (reduced glutathione) and fatty acids, and the intermediate product R5P was converted into fructose-6-phosphate and GA3P for glycolysis or purine nucleotide synthesis (Payen et al., 2016). Over-activated PPP increased oxygen consumption and made glioma cells more sensitive to hypoxia-induced death (Thiepold et al., 2017). Therefore, irreversible activating mTORC1 to forcibly drive PPP, while obstructing the glycolysis may control gliomas by inducing hypoxic damage.

### Lipid Metabolism

As building materials and energy sources, lipids are essential for glioma cells. Exogenous lipids were mainly obtained from intracranial glial cells in the form of lipoproteins through intercellular exchange, but rarely from the periphery (An and Weiss, 2016). The low-density lipoprotein (LDL) receptor-mediated cholesterol uptake supported the survival of glioma cells, which was counteracted by ATP-binding cassette sub-family A member (ABCA)-1-dependent cholesterol efflux promoted by liver X receptor agonists (Villa et al., 2016). Even without cholesterol intake, glioma cells and GSCs can synthesize cholesterol *de novo*. Cholesterol was synthesized from acetyl-CoA through sterol regulatory element-binding protein (SREBP)-2 and mobilized from endoplasmic reticulum via sterol *O*-acyltransferase (SOAT) and stored in lipid droplets in the form of cholesterol ester (Geng et al., 2016). Lipid droplets then activated SREBP-1, which was overexpressed in malignant glioma cells and can initiate angiogenesis and the synthesis of lipids on cell membrane and organelle via fatty acid synthase (FASN) (Zhou et al., 2016). Increased levels of polyunsaturated fatty acid synthetase ELOVL2 (elongation of very long chain fatty acids protein) and cholesterol synthase 3-hydroxy-3-methylglutaryl-CoA reductase (HMGCR) were also found in GSCs, involved in the elongation of fatty acids, synthesis of membrane lipids and facilitation of EGFR signaling to support cell growth (Wang X. et al., 2017; Gimple et al., 2019), and the impediment of fatty acid activator fatty acyl-CoA synthetase VL3 decreased the expression of stem-like phenotype CD133 and self-renewal functional molecules aldehyde dehydrogenase, musashi-1 and SOX2 on GSCs (Sun et al., 2014). The brain fatty acid binding protein (B-FABP, FABP7) participating in the utilization of unsaturated fatty acids also acted as a risk factor to drive the migration and infiltration of glioma cells and the growth of GSCs (Elsherbiny et al., 2013; Morihiro et al., 2013) dependent on the ratio of arachidonic acid (AA) to docosahexaenoic acid (DHA) (Elsherbiny et al., 2018). Prostaglandin E2 (PGE2) is another unsaturated fatty acid and known as an inducer of inflammation and pain, which was catalyzed from AA by cyclooxygenase (COX)-2 and prostaglandin E synthase (PGES) overexpressed in glioma cells, especially mesenchymal cells (Behnan et al., 2019). After binding to their receptors (EPs) in glioma tissues, PGE2 promoted tumor growth, invasion and immune escape (Jiang et al., 2017), and induced the angiogenesis with 20-hydroxyeicosatetraenoic acid (20-HETE), a transformation product of AA mediated by cytochrome P450 4A (CYP4A) (Feng et al., 2017; Wang C. et al., 2019). Inhibition of EPs or application of NSAIDs hindered the growth of gliomas (Seliger and Hau, 2018; Qiu et al., 2019). The synthesis and utilization of fatty acids and cholesterol participated in the malignant progression of gliomas, which were controllable under AMPK blockade (Guo et al., 2009; Kim et al., 2018).

Similar to carbohydrate, lipids were also catabolized by glioma cells to obtain energy. Ketone bodies are intermediate products of fatty acid oxidation, which were transported to brain tissues and converted to acetyl-CoA for vital activities. Considering that brain tumors cannot use ketone bodies and rely on glucose, the ketogenic diet was developed to limit the energy supply of gliomas rather than normal tissues (Poff et al., 2019). However, recent studies have proved that glioma cells can oxidize ketone bodies via up-regulated monocarboxylate transporter (MCT) during ketogenic diet (De Feyter et al., 2016), suggesting the ability of glioma cells to gain energy from lipids. In addition to fast-cycling glioma cells dependent on aerobic glycolysis, a subpopulation of slow-cycling cells with lipid transport and oxidation as the main metabolic mode has been confirmed, which can obtain energy in the absence of glucose (Hoang-Minh et al., 2018). Under oxygen-rich conditions, fatty acid oxidation was an essential energy pathway for glioma cells expressing high levels of fatty acid oxidases such as carnitine palmitoyltransferase 1 to grow independently of glycolysis (Lin et al., 2017; Wu et al., 2019). Nevertheless, the energy-producing efficiency of fatty acid oxidation is far less than glycolysis. Conversion of glycolysis to fatty acid oxidation by activating PPARα eventually led to the depletion of ATP in glioma cells (Wilk et al., 2015).

Distinguishing from conventional lipid metabolism, lipid peroxidation is an over-oxidation of ROS and lipids in the cell membrane and cytoplasm, producing cytotoxic peroxides including malonaldehyde (MDA) and 4-hydroxy-2-nonenal (HNE). Although there is a potential correlation between lipid peroxidation and the grade of gliomas clinically (Atukeren et al., 2017), lipid peroxidation in differentiated glioma cells induced ferroptosis, a type of programmed death. To resist peroxidative damage, glioma cells initiate degradation of peroxidatively modified proteins through proteasome system (Nakayama et al., 2016), and abate ferroptosis by GSH, phospholipid hydroperoxidase glutathione peroxidase 4 (GPX4) and glutamate (Glu)/cystine (Cys) antiporter system Xc^–^. Depleting GSH and Cys or inhibiting system Xc^–^ and GPX4 impeded the survival of glioma cells and increased their sensitivity to radiation-induced lipid peroxidation (Wang et al., 2018; Ye et al., 2020).

Controlling lipid metabolism resisted the invasion of gliomas, such as the application of phytol, retinol, and quercetin acting on FASN and SREBP1/2 (Facchini et al., 2018; Damiano et al., 2019), and the inhibition of acetyl-CoA carboxylase 1 and HMGCR by oleic acid and hydroxytyrosol (Priore et al., 2017). PPARα activator fenofibrate also inhibited glioma growth by inducing the dependence of tumor cells on fatty acid oxidation instead of glycolysis (Wilk et al., 2015). On this basis, using fatty acid oxidation inhibitors such as etomoxir may limit the leftover energy-producing pathways of glioma cells (Lin et al., 2017; Petovari et al., 2018), which is a potential treatment strategy.

### Amino Acid and One-Carbon (C1) Metabolism

As synthesis materials or decomposition products of proteins, amino acids supported and regulated the growth of tumor cells and tumor stem-like cells (Mayers et al., 2016; Jones et al., 2018). The heterogeneous amino acid metabolism of glioma cells is formed during environmental adaptation. In order to eliminate ROS accumulation caused by vigorous glucose metabolism, the level of xCT, the light chain subunit of system Xc^–^ in glioma cells was up-regulated to promote the intake of Cys, providing raw material for the synthesis of GSH. This cytoprotective effect relied on glucose, whose deprivation rapidly depleted NADPH during ingestion of Cys, inducing cell death of GBM cells (Goji et al., 2017). Glu, another raw material of GSH, and its metabolite L-glutamine (Gln) are fuels for glioma growth, both of which can be autonomously synthesized by glioma cells or taken up from metabolites of astrocytes (Tardito et al., 2015). The bioenergy of conversion from Glu to Gln through Gln synthetase were provided by lactate produced during glycolysis of glioma cells and normal astrocytes. The glutaminase mediated the transformation from Gln to Glu, releasing amide nitrogen for the biosynthesis of purines and pyrimidines (Venneti and Thompson, 2017), which was accelerated by the generation of Gln via GSH (Tardito et al., 2015). The excessive secretion of Glu from glioma cells can trigger glioma-related seizures by binding to receptors on neurons around the tumor (Huberfeld and Vecht, 2016). Glu was also released from synaptic neurons as a neurotransmitter, initiating the cascade of AKT and MAPK signaling through the Glu receptor on the surface of glioma cells to promote invasion. Moreover, in a glucose-deficient condition, Glu were metabolized by Glu dehydrogenase (GLUD1)-1 into the intermediate product of TCA cycle, α-KG, which activated inhibitor of nuclear factor kappa-B kinase subunit β and nuclear factor κB (NF-κB) to promote glucose uptake by up-regulating GLUT1 (Wang X. et al., 2019). mTOR2 was activated by high levels of Gln (Liu B. et al., 2019) to regulate Glu/Gln metabolism, promoting Glu secretion, Cys uptake, GSH synthesis and Gln catabolism to obtain energy and transmit growth factor signaling for glioma cells by activating c-Myc (Gu et al., 2017).

As another critical part of amino acid metabolism, serine (Ser)/glycine (Gly) metabolism governs the synthesis of nucleotides, proteins and lipids, and is the hub of glycolysis and folate metabolism (Maddocks et al., 2017). Ser was synthesized from the glycolytic intermediate 3-phosphoglycerate via phosphoglycerate dehydrogenase (PHGDH), and was converted into Gly by mitochondrial serine hydroxymethyltransferase (SHMT2) (Kim D. et al., 2015; Venneti and Thompson, 2017). When Ser was sufficient, PKM2 was stimulated to promote glycolysis of glioma cells, while SHMT2 was activated to counteract augmented TCA cycle activity and save oxygen. Although Gly accumulation caused by SHMT2 was detrimental to cell growth, glioma cells expressed high levels of Gly decarboxylase (GLDC) to decompose Gly into innocuous metabolites, inhibition of which led to the loaded cytotoxic aminoacetone and methylglyoxal. Conversely, when Ser deficiency was sensed, glioma cells stopped cell cycle by activating cyclin-dependent kinase inhibitor p21 through p53 and promoted the synthesis of GSH to maintain survival (Venneti and Thompson, 2017).

Ser is the main source of C1 units during the conversion to Gly and the decomposition of Gly. Other amino acids including Glu, Gln, tryptophan (Trp), and methionine (Met) are also the source of C1 units. Glioma cells expressing high level of IDH3α up-regulated SHMT2 and facilitated the activation of cytosolic SHMT (SHMT1) to promote the release of C1 units (May et al., 2019). miR-940, which obstructs the folate cycle and C1 metabolism by inhibiting methylenetetrahydrofolate dehydrogenase, was also down-regulated in glioma cells (Xu et al., 2019). The abundant C1 units participate in the biosynthesis of nucleotide and produce CO2 and NADPH, enabling glioma cells to survive, proliferate and invade under hypoxic conditions (Wypych and Baranska, 2020). Furthermore, C1 units can be thoroughly utilized by GSCs with rich purine synthases (Wang Q. et al., 2017), promoting the onset and rapid recurrence of GBM.

For malignant invasion, glioma cells overexpressed amino acid metabolic enzymes to resist hypoxia and glucose deficiency. Based on the distinct metabolic characteristics of tumors and normal brain tissues, intervention of amino acid metabolism was a selective means to improve gliomas (Panosyan et al., 2017). Moreover, considering efficient Gln uptake in glioma tissues, PET technology has been developed for imaging, which overcame the limitations of conventional nuclide ^18^F-FDG in the context of normal brain tissues with similar strong capacity of glucose uptake (Venneti et al., 2015).

The metabolic reprogramming allowed glioma cells to proliferate regardless of the ischemic lesion (Table 1), and endowed them with strong migration capabilities for a better growth condition (Kathagen-Buhmann et al., 2018), enabling the rapid invasion into healthy brain tissues. Concurrently, to cope with changes in nutritional sources, the metabolic plasticity of glioma cells resulted in the resistance to anti-metabolic therapies, including diet and drugs (De Feyter et al., 2016; Shibao et al., 2018). It may be a forward therapeutic strategy to block the adaptively up-regulated metabolic enzymes and activating factors in glioma cells appropriately while limiting the intake of energy substrates.

**TABLE 1:** Metabolism processes of glioma cells and GSCs.

Process	Reported cell lines	Condition	Substrate	Key media	Significance
Glucose uptake (Wang et al., 2015; Xu et al., 2015; Zheng et al., 2016; Kuang et al., 2017; Lee J.H. et al., 2017; Lee et al., 2018; Libby et al., 2018)	GBM (U87, U251, A172, LN229, U343, T98G), astrocytoma (U373); GSC isolated from GBM patients	Extracellular glucose	Glucose	GLUT1/3	Ensure enough raw materials for glucose metabolism
Fructose uptake (Su et al., 2018)	GBM (LN229, U87)	Extracellular fructose	Fructose	GLUT5	Provide supplemental energy sources other than glucose
Fructose decompose (Gao et al., 2018)	GBM (LN229, U87)	Abundant substrate	Fructose	Ketohexokinase	Provide fructose-1,6-diphosphate for glycolysis in glucose deficiency
Glycolysis (Wang et al., 2015; Agnihotri and Zadeh, 2016; Kathagen-Buhmann et al., 2016, 2018; Lee J.H. et al., 2017; Lee et al., 2018; Liu B. et al., 2019)	GBM (U87, U251, G55, U118, A172, LN229, U343, T98G), astrocytoma (U373), GSC (GS-11, GS-12, BT112)	Hypoxia	Glucose	HK, PFK1, PKM	Ensure energy source in hypoxic lesions; Promote migration to healthy brain tissues; Promote the shift of carbon from glucose into R5P for nucleotide generation
PPP (Kathagen-Buhmann et al., 2016; Payen et al., 2016; Liu R. et al., 2019)	GBM (G55, U87)	Oxygen, and sufficient substrate produced by HK	G6P	6-phosphogluconate dehydrogenase	Adjust pH; Produce R5P for glycolysis or purine nucleotide synthesis; Produce NAPDH for the synthesis of GSH and fatty acids
OXPHOS (Duan et al., 2018; Shibao et al., 2018)	GBM (U251), GSC derived from murine neural stem/progenitor cells	Glucose deficiency with sufficient oxygen	Lactate	Transport: MCT1/4; Reaction: TCA cycle	Switch metabolic mode from glycolysis to resist glucose-deficient environment
Cholesterol uptake (An and Weiss, 2016; Villa et al., 2016)	GBM (U87, U251, T98, A172), astrocytoma (U373)	Extracellular cholesterol from glial cells	LDL	LDL receptor	Provide material for organelle formation
Cholesterol efflux (An and Weiss, 2016; Villa et al., 2016)	GBM (U87, U251, T98, A172), astrocytoma (U373)	Intracellular cholesterol	Cholesterol ester	ABCA1	Induce cell death when over-activated
Cholesterol synthesis and mobilization (Geng et al., 2016)	GBM (U87, U251, T98), tumor cells isolated from GBM patients	Abundant substrate from glycolysis and fatty acid oxidation	Acetyl-CoA	Synthesis: SREBP-2, HMGCR; Mobilization: SOAT	Involve in the formation of cell membranes; Trigger SREBP-1-mediated lipid synthesis
Fatty acid synthesis and elongation (Geng et al., 2016; Zhou et al., 2016; Gimple et al., 2019)	GBM (U87, U251, T98), tumor cells and GSCs isolated from GBM patients	Intracellular glucose or cholesterol on ER membrane	Glucose, acetyl-CoA, cholesterol	SREBP-1, FASN, ELOVL2	Provide lipids on cell membrane and organelle
Unsaturated fatty acid utilization (Morihiro et al., 2013; Feng et al., 2017; Elsherbiny et al., 2018; Wang C. et al., 2019)	GBM (U87, U251, U373, M049, M103, M016), GSC (G144), astrocytoma (C6), GSC isolated from GBM patients	Abundant AA and less DHA	AA	B-FABP, COX-2, PGES	Provide eicosanoids and PGE2 for tumor growth, infiltration, immune escape and angiogenesis
Ketone body uptake and oxidation (De Feyter et al., 2016)	GBM (RG2); gliosarcoma (9L)	Ketogenic diet	Ketone bodies	Uptake: monocarboxylate transporter; Oxidation: hydroxybutyrate dehydrogenase	Resist the energy limitation of diet therapy
Fatty acid oxidation (Wilk et al., 2015; Lin et al., 2017)	GBM (U87, LN-229), tumor cells and GSCs isolated from GBM patients	Sufficient oxygen	Fatty acids	Carnitine palmitoyltransferase 1	Obtain energy in the absence of glucose (far less efficient than glycolysis)
Lipid peroxidation (Nakayama et al., 2016; Wang et al., 2018)	GBM (U87, U251, U373, SHG-44), astrocytoma (C6)	Depletion of GSH and Cys	Organelle lipids	ROS	Produce cytotoxic peroxide and induce cell death
Cys uptake (Goji et al., 2017)	GBM (U251, T98, A172, LN229)	Abundant glucose	Cys	System Xc^–^	Provide synthetic raw materials for antioxidant GSH
Glu uptake and Glu/Gln generation (Tardito et al., 2015)	GBM (U87, U251, LN229, LN18, SF188, GUVW)	Gln starvation or abundant Gln	Glu (uptake); Glucose and alanine (*de novo* synthesis); Glu and Gln (mutual conversion)	Uptake: excitatory amino acids transporters; Synthesis: pentose phosphate pathway, glycolysis and TCA enzymes, ALT; Mutual conversion: glutaminase/Gln synthetase	Provide synthetic raw materials for antioxidant GSH; Trigger glioma-related seizures; Provide amide nitrogen for synthesis of purines and pyrimidines during transformation from Gln to Glu
Glu oxidative deamination (Wang X. et al., 2019)	GBM (U87, U251, LN18), GSC (GSC11)	Glucose deficiency	Glu	Glutamate dehydrogenase 1	Replenish α-ketoglutarate for TCA cycle; Up-regulate GLUT1 and promote glucose uptake
Ser synthesis (Kim D. et al., 2015)	GBM (U251, LN229, 0308, BT145)	Abundant substrate from glycolysis	3-phosphoglycerate	Phosphoglycerate dehydrogenase	Promote glycolysis, activation of TCA cycle and oxygen saving
Conversion of Ser to Gly (Kim D. et al., 2015)	GBM (U251, LN229, 0308, BT145)	Abundant substrate	Ser	SHMT2	Release C1 units; Produce cytotoxic aminoacetone and methylglyoxal
Gly decarboxylation (Kim D. et al., 2015)	GBM (U251, LN229, 0308, BT145)	Gly loading	Gly	Glycine decarboxylase	Convert Gly into non- cytotoxic metabolites
C1 unit release (May et al., 2019)	GBM (U87, LNZ308), GSC (GIC-20, GIC-387)	Functional IDH3α expression	Ser	SHMT2/SHMT1	Provide synthetic raw materials for nucleotide
Nucleotide synthesis (Wypych and Baranska, 2020)	Astrocytoma (C6)	Abundant substrate and carrier	C1 units, amino acids, R5P, CO2	*De novo* synthetase, remedial synthase, tetrahydrofolate (carrier)	Promote survival, proliferation and invasion

## Glioma Cell Metabolism Links to Immune Cells in Microenvironment

**FIGURE 2:**
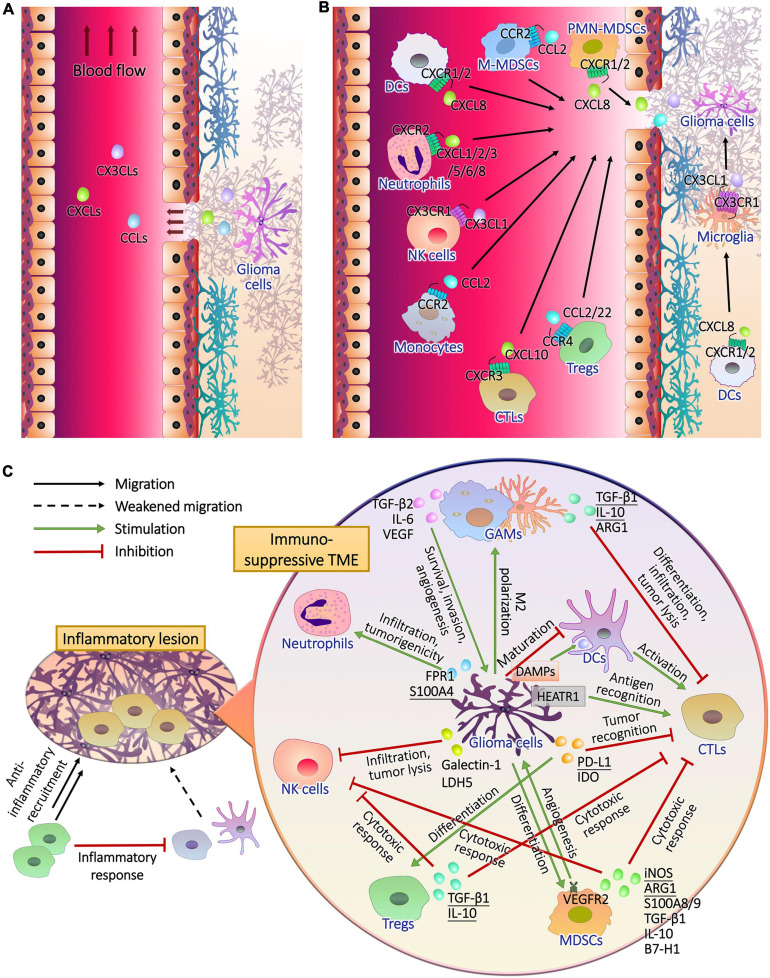
The recruitment of immune cells and the formation of immunosuppressive glioma microenvironment. **(A)** Glioma cells release chemokines to lesion tissues, part of which entered peripheral blood through the pathological blood brain barrier. **(B)** After the chemokine receptors of peripheral and intracranial immune cells captured their ligands, they drive the cells to migrate upstream where chemokines are released. **(C)** During the infiltration of inflammatory T cells, anti-inflammatory cells, mainly regulatory T cells (Tregs), are recruited to inhibit the antigen presentation of dendritic cells (DCs) and T cell activation, resulting in impaired immune response. In addition to the activation of anti-inflammatory mechanisms, glioma cells perform complex intercellular interactions with immune cells in tumor microenvironment. Cytotoxic T lymphocytes (CTLs) should recognize tumor antigen HEAT repeat-containing protein 1 (HEATR1) and kill glioma cells with natural killer (NK) cells, and CTLs were activated by mature DCs during the presentation of damage associated molecular patterns (DAMPs) released by glioma cells. Glioma cells inhibited the maturation of DCs. Furthermore, glioma cells secreted cytokines to induce the generation and recruitment of tumorigenic Tregs, myeloid-derived suppressor cells (MDSCs), neutrophils and M2 polarized microglia and macrophages, and inhibit the infiltration and tumor lysis of CTLs and NK cells.

**FIGURE 3:**
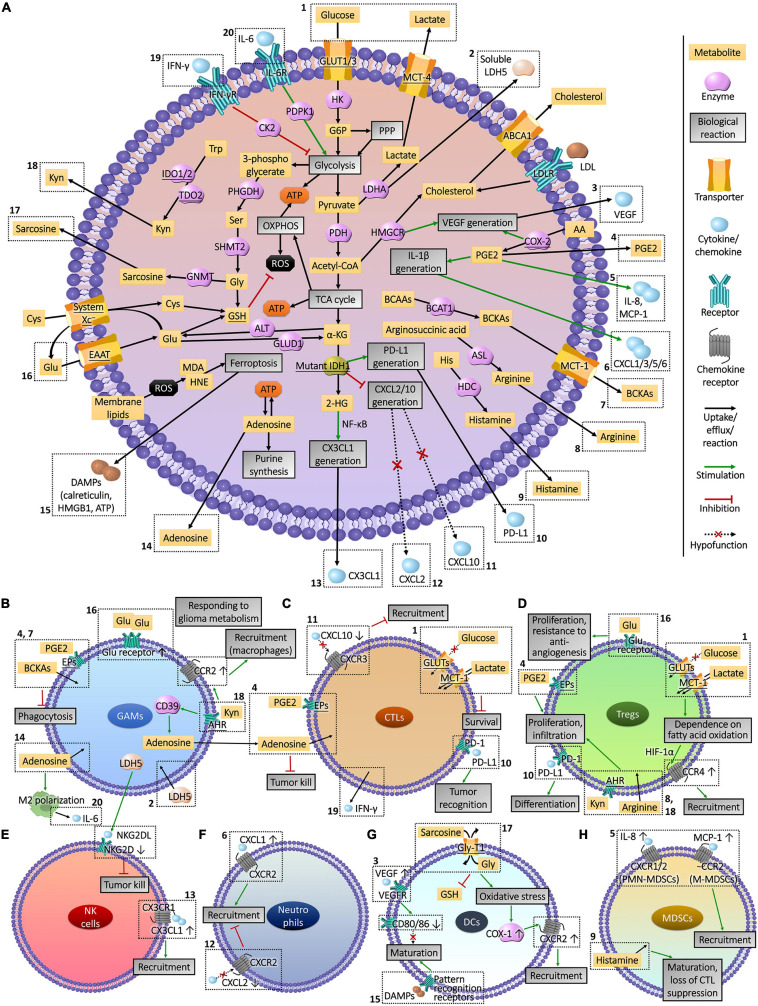
Interactions between glioma cell metabolism and immunomicroenvironment. **(A)** Metabolic reprogramming enables glioma cells to express high levels of substrate transporters and metabolic enzymes to obtain sufficient energy in the harsh conditions of lesion, resulting in the deprivation of nutrient substrates and accumulation of immune-interfering metabolites in the extracellular fluid. Abnormally expressed or mutated metabolic enzymes also affected immune cells by regulating the production of chemokines and other cytokines. The actions of these molecules that enter the tumor microenvironment on immune cells and the influences of immune cells on glioma cells metabolism are presented in part **(B–H)**. The Arabic numerals in the figure link the metabolism of glioma cells **(A)** with immune cells **(B–H)**.

Metabolic plasticity not only promoted the energy supply and the synthesis of substrates required for growth and heredity of glioma cells, but also induced immune evasion (Ganapathy-Kanniappan, 2017). Immune cells accumulating and infiltrating in the glioma tissues include glioma-associated microglia/macrophages (GAMs), T lymphocytes, natural killer (NK) cells, neutrophils, dendritic cells (DCs) and myeloid-derived suppressor cells (MDSCs) (Magana-Maldonado et al., 2016; Gieryng et al., 2017; Figures 2A,B), supporting tumor growth instead of surveillance and annihilation and limiting the prognosis (Zhang et al., 2017; Boussiotis and Charest, 2018; Figure 2C). Metabolic remodeling increased the level of metabolites from glioma cells to induce immune tolerance in the TME (Kesarwani et al., 2017), and drove the production of immunosuppressive factors such as arginase (ARG)-1, IL-10, and TGF-β (Guo et al., 2018; Figure 3A). Moreover, the hypoxia caused by uncontrolled proliferation of metabolic reprogrammed glioma cells reduced the viability of tumor killer cells, further facilitating the survival of glioma cells (Colwell et al., 2017). The interactions between glioma cell metabolism and immune cells are a novel perspective for understanding the immune escape and refractoriness of gliomas.

### GAMs

GAMs account for 30–50% of glioma-infiltrating immune cells, which is the highest proportion in tumor tissues (Hambardzumyan et al., 2016). Microglia were thought to be macrophages settled in the central nervous system (Sankowski et al., 2019), but their origins and phenotypes are different. Microglia originated from the neuroepithelial yolk sac progenitor cells (Gomez Perdiguero et al., 2015) with high level of CX3CR-1, but low level of CD45 and no CCR-2 expression (Hutter et al., 2019). In contrast, macrophages in glioma TME with CX3CR1 expression were differentiated from CX3CR1^lo^ peripheral monocytes entered the cranial cavity (Quail and Joyce, 2017), which highly express CD45 and CCR2 (Hambardzumyan et al., 2016; Chen Z. et al., 2017). Monocyte chemotactic protein 1 (MCP-1, CCL2) secreted by glioma cells mediated the recruitment of CCR2^+^ monocytes and macrophages (Chen Z. et al., 2017; Vakilian et al., 2017), and CX3CL1 induced the infiltration of CX3CR^+^ microglia (Hambardzumyan et al., 2016). Tumor recognition and phagocytosis functions of these innate immune cells were declined in glioma milieu (Poon et al., 2017), and cytokines such as TGF-β1 and IL-10 they secreted contributed to the formation of immunosuppressive TME (Roesch et al., 2018).

There is an interaction between GAMs and metabolites from glioma cells. Compared to homologous cells not exposed to the gliomas, up-regulated Glu receptors and GS and decreased xCT were detected in GAMs in response to Glu secreted by tumor cells (Choi et al., 2015). Meanwhile indoleamine 2,3-dioxygenase (IDO)-1/2 and tryptophan 2,3-dioxygenase (TDO)-2 were highly expressed in glioma cells and were proportional to tumor grade (Guastella et al., 2018), which catalyzed the decomposition of Trp into kynurenine (Kyn), a ligand of the aryl hydrocarbon receptor (AHR). The released Kyn induced the expression of CCR2 by activating AHR, and advanced the recruitment of macrophages to tumor sites by enhancing the response to MCP-1 secreted by glioma cells (Takenaka et al., 2019). In addition to the impact on recruitment, metabolism of glioma cells was involved in the regression of innate immune abilities. Branched-chain ketoacids (BCKAs) metabolized from branched-chain amino acids (BCAAs) and unsaturated fatty acid PGE2 released by glioma cells were taken up by GAMs, accompanied by decreased phagocytosis (Ghosh et al., 2010; Silva et al., 2017). On the other hand, Kyn secreted by glioma cells activated AHR in GAMs to inhibit the cytotoxicity of T lymphocytes by up-regulating the production of ectonucleotidase CD39 and adenosine (Takenaka et al., 2019). The polarization of GAMs to immunosuppressive M2 type is a representative tumorigenic process positively bound up with the grading and rapid recurrence of gliomas (Wang Q. et al., 2017; Sorensen et al., 2018), which was widely found in mesenchymal GBM cells and was related to NF1 loss (Wang Q. et al., 2017). M2 polarization was also induced by metabolites of glioma cells such as adenosine (Komohara et al., 2008; Kesarwani et al., 2019), along with the secretion of CSF-1 (Pyonteck et al., 2013), the expression of CCR5 (Laudati et al., 2017), and DNA damage repair (Meng et al., 2019) of glioma cells. However, GAMs polarized into immune-promoting M1 type were not facilely affected by metabolites (Guan et al., 2017). In addition to immunosuppressive functions, GAMs promoted the migration, angiogenesis, and invasion of gliomas via TGF-β2, IL-6, and VEGF (Roesch et al., 2018), which were affected by glioma cell metabolism as well. The secretion of VEGF and TGF-β from GAMs were reduced by blocking CYP4A to inhibit the synthesis of unsaturated fatty acid 20-HETE (Wang C. et al., 2017). The metabolism of glioma cells was also regulated by GAMs. IL-6 secreted by M2 macrophages promoted glycolysis of GBM cells by phosphorylating PGK1 via 3-phosphoinositide-dependent protein kinase 1 (PDPK1) (Zhang et al., 2018), and quinolinic acid secreted by microglia was taken up by glioma cells for the synthesis of NAD^+^ to resist oxidative stress (Sahm et al., 2013).

Glioma-associated microglia/macrophages are body guards of gliomas from the host. The metabolism of glioma cells is closely related to the recruitment, infiltration, and polarization of GAMs (Figure 3B). Utilizing the communications between glioma cells and GAMs can create the possibility to kill gliomas at close range, such as proper interventions in metabolism.

### T Lymphocytes

CD8^+^ cytotoxic T lymphocytes (CTLs) and CD4^+^ CD25^+^ FOXP3^+^ regulatory T cells (Tregs) are active lineages of glioma-infiltrating T lymphocytes. CTLs are the main contributors that infiltrate and kill tumor cells. In addition to the secretion of tumor-damaging cytokines such as IFN-γ, CTLs can recognize antigens such as HEAT repeat-containing protein 1 (HEATR1) expressed by glioma cells via human leukocyte antigen (HLA)-A2 [or major histocompatibility complex class 1 in animals] and trigger cell lysis (Wu et al., 2014; Shao et al., 2017). Due to less chance of the contact between ER and mitochondria, GSCs expressed low levels of sialylated glycans on cell surface and were more sensitive to CTLs than differentiated glioma cells (Bassoy et al., 2017). As part of TME, the cytotoxicity of CTLs cannot function properly. In response to the IFN-γ secreted by CTLs, glioma cells released PD-L1 through exosomes to interact with T cells for immune escape (Qian et al., 2018). IDH1/2 mutations reduced the release of CXCL10 that attract the accumulation of CTLs (Kohanbash et al., 2017), and increased the production of PD-L1 (Berghoff et al., 2017), hinting the involvement of glioma cell metabolism in the dysfunction of CTLs.

Glioma cells with heterogeneous carbohydrate and amino acid uptake capacity occupied the supply of glucose and amino acids in hypoxic niduses, leading to the exhaustion of CTLs (Mirzaei et al., 2017; Rashidi et al., 2020), and the accumulation of lactate during glycolysis destroyed the intracellular and extracellular concentration gradients, which hindered lactate efflux and reduced the viability of CTLs (Shao et al., 2017). As an immune checkpoint (Kesarwani et al., 2018), the Trp metabolism of glioma cells also limited the infiltration of CTLs through IDO1 (Zhai et al., 2018). In contrast, the histidine (His) metabolism promoted the activation of CTLs, and the decline of His decarboxylase (HDC) activated the inhibition of CTL infiltration by MDSCs (Ahn et al., 2015). The elevated levels of other metabolic enzymes including argininosuccinate lyase (ASL), ARG2, and COX-2 in glioma cells led to augmented synthesis of Trp, arginine and PGE2, which induced the repression of CTLs as well (Eberstal et al., 2014; Authier et al., 2015; Kesarwani et al., 2019). CTLs also affect the metabolism of glioma cells through IFN-γ, which activated casein kinase (CK)-2 of tumor cells to hold up glycolysis, impeding the growth of gliomas (Ghildiyal and Sen, 2017).

Treg is a T cell subtype responsible for anti-inflammatory and immune tolerance, secreting high levels of inhibitory cytokines including TGF-β and IL-10 (Qiu et al., 2020), which allowed glioma cells to escape the cytotoxic damage of CTLs in TME (See et al., 2015). In malignant gliomas, Tregs were associated with the tumor recurrence, resistance to targeted drugs and decrease of survival period (Sayour et al., 2015; Du Four et al., 2016), whose depletion improved the condition of glioma mice by inducing spontaneous rejection of the tumor. Glioma cell metabolism also affects Tregs. Augmented production of Trp and arginine, and elevated expression of metabolic enzymes IDO1, ASL and ARG2 in glioma cells were accompanied by increased infiltration of Tregs (Kesarwani et al., 2019). Mutations in the metabolic enzyme IDH1/2 promoted the secretion of PD-L1 (Berghoff et al., 2017), which induce the differentiation of Tregs (DiDomenico et al., 2018). Differentiated Tregs recruited into tumor region were mediated by chemokines MCP-1 and CCL22 secreted by glioma cells (Jacobs et al., 2010; Chang et al., 2016). Due to the efficient glucose uptake and glycolysis, glucose was deprived in the hypoxic lesions by glioma cells, resulting in Tregs relying on fatty acids for mitochondrial metabolism and migrating to glioma tissues in response to CCL22 in a HIF-1α-dependent manner (Miska et al., 2019). Accumulated lactate further drove the infiltration of Tregs, and the depletion of lactate from glioma cells decreased tumor-infiltrating Tregs (Chirasani et al., 2013). PGE2 synthesis in glioma cells with up-regulated COX-2 were also related to the dilation and infiltration of Tregs (Rolle et al., 2012; Authier et al., 2015). In addition, glioma cells can activate Tregs by enhancing extracellular transport of Glu to survive anti-angiogenic therapy (Long et al., 2020).

CTLs and Tregs are T cell lineages with opposite effects in glioma TME, which were both influenced by the metabolic state of cancer cells (Figures 3C,D). The intervention of glioma cell metabolism can promote the infiltration of CTLs and restore their anti-tumor immunity, meanwhile hinder the recruitment of Treg, relieving the immune tolerance in TME.

### NK Cells

Ly6c^+^ NK cells in the glioma TME are a group of innate tumor killer cells derived from the bone marrow, which are part of glioma-infiltrating lymphocytes. Due to different metabolic properties, the grade of gliomas was inversely proportional to the infiltration and anti-tumor functions of NK cells. Attributing to the recruitment inhibitor galectin-1 secreted by HGG cells (Baker et al., 2016), low levels of infiltrating NK cells were observed (Domingues et al., 2016; Zhu et al., 2019), which possessed faint cytotoxicity and expressed high level of Tim-3 to prevent the tumor-killing helper T cells type 1, shortening the survival time of patients (Pereira et al., 2018). Conversely, mutations of metabolic enzyme IDH1 in LGG cells produced 2-HG and activated NF-κB to promote the secretion of CX3CL1, which attracted CX3CR1-expressing NK cells to infiltrate, making patients with a good prognosis (Ren et al., 2019). The decreased immune functions of NK cells were also related to the metabolism of glioma cells. Soluble lactate metabolic enzyme LDH5 secreted by glioma cells induced tumor infiltrating myeloid cells and circulating monocytes to release the ligand of NK group 2 member D (NKG2D), a surface receptor on NK cells in a long term, leading to the down-regulation of NKG2D and malfunction of tumor lysis (Crane et al., 2014; Figure 3E). According to the grade of gliomas, a suitable metabolic intervention scheme should be adjusted to effectively exert the tumor infiltration and cytotoxicity functions of NK cells.

### Neutrophils

High proportion of neutrophils expressing CD11b and Ly6G in glioma tissues increased the tumor malignancy (Spiegel et al., 2016), becoming a powerful indicator of poor prognosis (Massara et al., 2017; Zhang et al., 2017). The S100A4 expressed in GSCs, which is a novel biomarker promoting the transcription of genes involving glycolysis and gluconeogenesis (Chow et al., 2017), induced tumorigenicity of neutrophils involving the promotion of tumor growth, metastasis, and resistance to anti-angiogenic drugs (Liang et al., 2014). Besides, metabolism of glioma cells interfered the recruitment and infiltration of neutrophils (Figure 3F). Neutrophils were recruited to the periphery of the glioma inflammatory region via chemokines including CXCL1/2/3/5/6 and IL-8 (CXCL8), which were secreted from glioma cells due to the expression of IL-1β (Lee S.Y. et al., 2017; Mostofa et al., 2017). Subsequently, neutrophils infiltrated the core area of tumor tissues through formylpeptide receptor 1 (FPR1) secreted by GBM cells to promote tumor growth, invasion and angiogenesis (Liu et al., 2012). The expression of COX-2 in glioma cells promoted the anabolic metabolism of PGE2, up-regulating the expression of IL-1β and CXCL1 (Jiang and Dingledine, 2013) to recruit neutrophils (Mostofa et al., 2017). The IDH1 mutation in glioma cells weakened the CXCL2-mediated recruitment of neutrophils (Amankulor et al., 2017), which may be one of the reasons for the low malignancy of LGGs. Despite the pro-glioma effects, utilizing the performance of directional migration and infiltration to the tumor area through BBB, the localized drug delivery and imaging tracer based on neutrophil carrier raised the treatment and staging diagnosis of glioma to a new height (Osuka and Van Meir, 2017; Xue et al., 2017; Wu et al., 2018), and can inspect the efficacy of metabolic interventions.

### Dendritic Cells

As resident antigen presenting cells (APCs) in central nervous system, DCs possess the ability to activate glioma-killing CTLs (Malo et al., 2018a). The decrease of tumor-infiltrating DCs led to reduced survival of GBM mice (Mathios et al., 2016). DC vaccine-based immunotherapy has been widely investigated in the treatment of gliomas (Mitchell et al., 2015; Weller et al., 2017). However, the anti-tumor functions of DCs were covered by the glioma-induced immunosuppression, which limited the effectiveness of DC therapy (Garg et al., 2016; Li et al., 2018). Glioma cells recruited DCs through the CXC chemokine family (Dastmalchi et al., 2019). Under the exposure to glioma cells expressing VEGF, the maturation of DCs were suppressed and antigen presentation and T-cell activation capabilities were diminished (Sheng et al., 2020). What’s more, DCs in the glioma TME exhibited tumor tolerable properties, expressing more IDO to induce Tregs to infiltrate glioma tissues for immune escape (Wainwright et al., 2014; He et al., 2015). The decrease of immunosuppressive glioma-infiltrated DCs via immune checkpoint inhibitors improved T cell responses and survival of GBM mice (Hung et al., 2018).

The recruitment of DCs was affected by amino acid metabolism of glioma cells (Figure 3G). Glycine-*N*-methyl transferase mediates the conversion of Gly to sarcosine, which released from glioma cells and competed with DCs for Gly uptake through glycine transporter type-1 (Gly-T1) (Dastmalchi et al., 2019). Gly depletion led to decreased GSH and oxidative stress of DCs, leading to the up-regulation of COX-1 to promote CXCR2 expression and responding to IL-8 from glioma region. The immune tolerance and malignant transformation of DCs were also related to glioma cell metabolism. By inhibiting glycolysis or LDHA, the proliferation, migration and infiltration into glioma tissues of malignantly transformed DCs were repressed (Sheng et al., 2020), and these DCs secreted high levels of IL-12 to induce anti-tumor behavior of T cells (Chirasani et al., 2013). In addition, DCs stayed in immature state and co-stimulatory molecules CD80 and CD86 were down-regulated owing to elevated secretion of VEGF from glioma cells (Malo et al., 2018b) expressing elevated metabolic enzymes (He et al., 2015), involving HK2, PHGDH (Wolf et al., 2011; Liu et al., 2013), HMGCR (Slawinska-Brych et al., 2014), COX-2 (Feng et al., 2017), nitric oxide (NO) metabolic regulation enzyme dimethylarginine dimethylaminohydrolase (Boult et al., 2011) and mutated IDH1 (Wang et al., 2014).

The loss of immunogenicity of tumor cells is a cause of the failure of APCs. Inducing the release of damage associated molecular patterns (DAMPs) from glioma cells restored their immunogenicity, and triggered the activation of CTLs by DCs (Dastmalchi et al., 2019). Lipid peroxidation and ferroptosis induced by photodynamic therapy induced dying glioma cells to release DAMPs such as calreticulin, high mobility group protein B1 (HMGB1) and ATP that can be swallowed by DCs, stimulating DC maturation and activation (Turubanova et al., 2019). It can be inferred that the restoration of antigen recognition and presentation abilities of glioma-infiltrating DCs via the domination of glioma cell metabolism is critical to their anti-glioma functions, which could authentically exert the efficacy of DC vaccine on gliomas.

### Myeloid-Derived Suppressor Cells

Myeloid-derived suppressor cells are a lineage of immature bone marrow-derived cells (BMDCs) activated under pathological conditions, including CD11b^+^ Ly6C^hi^ Ly6G^–^ immature monocytes (M-MDSCs) and CD11b^+^ Ly6C^lo^ Ly6G^+^ immature polymorphonuclear cells (PMN-MDSCs, also known as G-MDSCs, immature granulocytes) (Marvel and Gabrilovich, 2015). Regardless of their origins, the monocyte marker HLA-DR is hardly expressed in M-MDSCs, while PMN-MDSCs express lectin-type oxidized LDL receptor 1, which is hardly detected in neutrophils (Gabrilovich, 2017). MDSCs account for more than 40% of glioma-infiltrating immune cells (Kamran et al., 2017) and expressed IL-4Rα, inducible nitric oxide synthase (iNOS) and ARG1 to inhibit the responses of glioma-killer T lymphocytes and NK cells (Kohanbash et al., 2013; Gielen et al., 2016), causing patients poor prognosis (Alban et al., 2018). Among MDSCs, PMN-MDSCs induced CD4^+^ glioma-infiltrating T lymphocytes to express PD-1, exhibiting more prominent T cell suppression (Dubinski et al., 2016). Furthermore, the expression of VEGFR2 in MDSCs promoted the malignant progression of gliomas by inducing angiogenesis (Huang et al., 2017).

The generation, infiltration, and acquisition of immunosuppressive capacity of MDSCs were regulated by the metabolism of unsaturated fatty acids and amino acids of glioma cells (Figure 3H). When exposed to the exosomes released by glioma cells, the expansion of BMDCs with MDSCs phenotype was induced in healthy BMDCs (Gielen et al., 2016; Guo et al., 2019), secreting IL-10, TGF-β, Fas-ligand and B7-H1 to inhibit the activation of T lymphocytes (Chae et al., 2015). The development of ARG1 phenotype in MDSCs was also induced by GSCs in a CXCR2-dependent manner, via the secretion of macrophage migration inhibitory factor (Otvos et al., 2016). Then, M-MDSCs and PMN-MDSCs migrated to TME in response to MCP-1 and IL-8 released from glioma cells separately, which can recruit monocytes and neutrophils expressing same chemokine receptors, CCR2 or CXCR1/2 (Chang et al., 2016; Ding et al., 2019). Anabolism of PGE2 in glioma cells was involved in the generation and recruitment of M-MDSCs and PMN-MDSCs. Depending on the level of COX-2 (Fujita et al., 2011; Kosaka et al., 2014), PGE2 promoted the secretion of IL-8 from glioma cells through autocrine (Venza et al., 2011, 2012), advanced the migratory response of M-MDSCs to MCP-1, and promoted the expression of ARG1 in PMN-MDSCs. However, histamine secreted from glioma cells mediated the maturation of MDSCs and the loss of immunosuppressive functions. The exhaustion of histamine via HDC knockout resulted in augmented infiltration of MDSCs into glioma tissues with suppressed CTLs (Ahn et al., 2015). Studying the relationship between the generation and recruitment of MDSCs and glioma cell metabolism is expected to improve the immune microenvironment of the lesions.

## Metabolic Immunotherapy Strategies for Glioma

Metabolic heterogeneity of tumor cells and their initiating cells promoted the rapid invasion and recurrence of gliomas (Kathagen-Buhmann et al., 2016; Shibao et al., 2018). In the hypoxic area of intracranial lesions, these malignant cells made thorough use of glucose, lipoproteins and amino acids to ingest energy, synthesize hereditary substance, and produce antioxidants to resist oxidative stress. Metabolic reprogramming also brought enhanced migratory ability for glioma cells to invade healthy brain tissues for more energy supply. In order to ensure the smoothness of these biological processes, glioma cells took advantage of their metabolic properties for immune tolerance and escape mainly through the following ways.

(1) Their powerful nutrient uptake depleted the energy supply of tumor killer cells, and enhanced the synthesis of immune checkpoints including PD-L1 and IDO, impairing the anti-tumor responses.
(2) The metabolites secreted into TME promoted the infiltration and expansion of inflammatory neutrophils and suppressive Tregs and MDSCs to activate

anti-inflammatory mechanisms and immunotolerant responses.
(3) The metabolites led to malignant transformation of tumor-infiltrating APCs and phagocytes and exerted immunosuppressive functions, indirectly resisting tumor killer cells.
(4) After metabolic remodeling, the ability to recruit immune-tolerant cells was enhanced, but anti-tumor cells were less recruited. On the contrary, peroxidation metabolism enhanced immunogenicity and attract the presentation of tumor antigens (Turubanova et al., 2019).

Energy production and antioxidant responses during metabolism of glioma cells can also be induced by GAMs (Sahm et al., 2013; Zhang et al., 2018), but inhibited by CTLs in TME (Ghildiyal and Sen, 2017), reflecting the bidirectional conversation between glioma cell metabolism and immune microenvironment.

Several strategies for improving glioma TME through metabolic therapy are recommended (Table 2). Glycolysis inhibitors, or drugs targeting GLUT3 and MCT4 can restore the supply of extracellular glucose and reduce the lactate stress, improving the growth of CTLs, and hindering the tendency of Tregs to fatty acid oxidation to inhibit the infiltration of Tregs. Inhibiting inflammation and the release of PGE2 by anti-COX drugs such as NSAIDs can hinder inflammatory infiltration and activate anti-inflammatory mechanisms, restore the phagocytosis of GAMs and tumor lysis of CTLs, and repress the recruitment of Tregs, neutrophils and MDSCs. Inhibiting key enzymes in Trp–Kyn pathway and adenosine synthesis to prevent the loaded Kyn from activating AHR of GAMs and Tregs, and reduce the concentration of adenosine to enhance the cytotoxicity of CTLs is also a good choice. Compounds designed to act on amino acid transporters, including MCT1 and system Xc^–^ can inhibit the release of BCKAs and the uptake of GSH synthetic materials, so as to restore GAMs phagocytosis, and induce the release of glioma-derived DAMPs recognized by DCs during ferroptosis. In addition, local injection of sarcosine and histamine can promote the migration of DCs to glioma region and promote the maturation of MDSCs. For glioma cells with IDH mutations, targeting mutant IDH can restore the release of CXCL10 and inhibit the production of PD-L1, promoting recruitment and tumor recognition of CTLs and inhibiting Tregs. It can be combined with imaging methods such as PET to analyze the metabolic characteristics of the lesions (Venneti et al., 2015) and formulate an individualized metabolic treatment plan.

**TABLE 2:** The impact of metabolic remodeling of glioma cells on immune cells.

Altered metabolic media	Metabolic characteristics after remodeling	Influence on immune cells in glioma TME	Significance	Treatment strategies
Upregulated GLUT1/3, amino acid transporters and glycolytic enzymes	Increased glucose and amino acid uptake and activation of glycolysis	Deprive nutrients and accumulate intracellular lactate in CTLs to deplete CTLs	Block tumor lysis (Mirzaei et al., 2017; Shao et al., 2017; Rashidi et al., 2020)	Glycolysis inhibitors (targeting HK, PFK-1, PKM), glucose and amino acid uptake inhibitors (targeting GLUT1/3 and MCT4), and ketogenic diet
		Induce Tregs to survive on fatty acid oxidation and promotes CCR4 expression by activating HIF-1α	Promote immunosuppressive recruitment (Miska et al., 2019)	
		Enhance lactate uptake of macrophages and DCs through MCT1 to induce the malignant transformation of macrophages and DCs	Induce the tumorigenicity of immune cells (Sheng et al., 2020)	
IDH1/2 mutations	Conversion of α-KG to 2-HG in TCA cycle	Inhibit the generation of CXCL10 to prevent the infiltration of CTLs	Block tumor lysis (Kohanbash et al., 2017)	Mutant IDH brain-targeted inhibitors (AG120, AG221, AG881) (Fujii et al., 2016)
		Induce glioma cells to secrete PD-L1 to inhibit the antigen recognition of CTLs and promote the differentiation of Tregs	Suppress anti-tumor response and promote immune suppression (Berghoff et al., 2017)	
		Activate NF-κB to promote the generation of CX3CL1 to promote the infiltration of NK cells	Promote anti-tumor infiltration in low-grade gliomas (Ren et al., 2019)	
		Inhibit the generation of CXCL2 to prevent the infiltration of neutrophils	Suppress the infiltration of tumorigenic cells (Amankulor et al., 2017)	
		Increase VEGF generation to reduce the expression of co-stimulatory molecule in DCs and hinder the maturation	Prevent antigen presentation to inhibit anti-tumor response (Wang et al., 2014; Malo et al., 2018b)	
Increased Trp synthesis and up-regulated IDO1	Increased synthesis of Kyn	Activate AHR of macrophages and increase CCR2 expression to promote the recruitment of macrophages	Promote the infiltration of tumorigenic cells (Takenaka et al., 2019)	IDO1/2 and TDO2 inhibitors
		Up-regulate CD39 to promote adenosine synthesis by GAMs to decrease the activity of CTLs	Suppress tumor lysis (Takenaka et al., 2019)	
		Activate AHR of Tregs to promote their proliferation and infiltration	Promote immunosuppressive infiltration (Kesarwani et al., 2019)	
Up-regulated COX-2 and PGES	Increased synthesis of PGE2	Impair the phagocytic activity of GAMs and the tumor lysis function of CTLs, and promote the proliferation and infiltration of Tregs by activating EPs	Suppress anti-tumor response and promote immunosuppressive infiltration (Ghosh et al., 2010; Kesarwani et al., 2019)	Drug uses NSAIDs as the lead compound
		Increase IL-1β transcription and CXCL1 generation to promote the recruitment of neutrophils	Promote the infiltration of tumorigenic cells (Jiang and Dingledine, 2013; Mostofa et al., 2017)	
		Increase IL-8 and MCP-1 generation to promote the recruitment of MDSCs	Promote immunosuppressive infiltration (Venza et al., 2011, 2012)	
		Increase VEGF generation to reduce the expression of costimulatory molecule in DCs and hinder the maturation	Prevent antigen presentation to inhibit anti-tumor response (Feng et al., 2017; Malo et al., 2018b)	
Up-regulated MCT1	Excessive generation and efflux of BCKAs from the catabolism of BCAAs	Impair the phagocytic activity of GAMs during the uptake and re-aminate of BCKAs	Block tumor phagocytosis (Silva et al., 2017)	MCT1 inhibitors
Overexpressed Cys/Glu transporter (xCT)	Mass release of Glu to the outside of cell	Up-regulate Glu receptors and GSH, down-regulate xCT in GAMs	Reflect the response of immune cells to glioma cell metabolism (Choi et al., 2015)	xCT inhibitors
		Activate and expand Tregs	Promote the resistance to anti-VEGF therapy (Long et al., 2020)	
Activated adenosine metabolism	Increased synthesis and release of adenosine	Promote M2 polarization of GAMs	Induce the formation of immunosuppressive cells (Kesarwani et al., 2019)	inhibitors targeting adenosine or its receptors
Up-regulated ASL and downregulated iNOS	Increased synthesis and inhibited catabolism of arginine	Induce the proliferation of GAMs and Tregs	Enhance immune suppression (Kesarwani et al., 2019)	ASL inhibitors and agonists targeting iNOS and creatine kinase mitochondrial 1
Generation of LDH5	Secretion of extracellular soluble LDH5	Induce GAMs and circulating monocytes to release NKG2DL to inhibit the cytotoxicity of NK cells by down-regulating NKG2D	Block tumor lysis (Crane et al., 2014)	LDH5 inhibitors
Up-regulated HK2, PHGDH, and HMGCR	Increased synthesis of Ser and cholesterol, and stimulated glycolysis	Increase VEGF generation to reduce the expression of costimulatory molecule in DCs and hinder the maturation	Prevent antigen presentation to inhibit anti-tumor response (Wolf et al., 2011; Liu et al., 2013; Slawinska-Brych et al., 2014; Malo et al., 2018b)	HK2, PHGDH and HMGCR inhibitors
Up-regulated GPX4 and system Xc^–^	Increased synthesis of GSH and inhibited lipid peroxidation	Block the release of DAMPs and prevent DCs from recognizing glioma cells	Inactivate CTL-mediated anti-tumor response (Li et al., 2018; Wang et al., 2018; Ye et al., 2020)	GPX4 and system Xc^–^ inhibitors

## Discussion

The microenvironment where glioma cells regulate immune cells through metabolic reprogramming is in the brain. Therefore, a challenge for glioma metabolic immunotherapy is the BBTB. In order to selectively act drugs on the lesions and reduce the impact on peripheral tissues, a brain drug delivery system needs to be established. Osmotic BBB disruption based on intra-arterial infusion of hypertonic mannitol solution, intravenous bradykinin analogs that relax tight junctions, drugs coupling to a mediator targeting and shuttling insulin or transferrin receptors, nanoparticle drug delivery system, and direct delivery of drugs to the brain parenchyma or excision cavity are potential methods for metabolic immunotherapeutic drugs to cross BBTB (van Tellingen et al., 2015; Li et al., 2020). Targeting metabolic functional proteins selectively and highly expressed in glioma cells, such as GLUT3 (Xu et al., 2015; Zheng et al., 2016), or using targeted metabolic drug delivery systems based on neutrophils (Osuka and Van Meir, 2017; Xue et al., 2017) or other immune cells accumulating in glioma tissues is also a good solution.

To conclude, glioma cells show different metabolic pattern and immune microenvironment from peripheral tumors (Table 3). Interfering with cell metabolism could not only hinder the growth of glioma cells, but also improve the immune response in the focal area to systematically resist tumor progression, which is expected to become a new direction for clinical treatment of gliomas.

**TABLE 3:** Differentials in cell metabolism and immune TME of gliomas compared with peripheral tumors.

What’s special	Specific performance	Significance
Richer energy substrate	Compared with peripheral tissues, brain has higher energy substrate requirement, especially for glucose (Aldana, 2019). The vascular endothelial cells in BBB highly express GLUT1 to take up abundant glucose from the periphery blood into the brain (Jais et al., 2016).	Glioma cells possess more energy substrates than peripheral tumor cells.
Utilization of Glu/Gln metabolic coupling of brain cells	Glu released from synaptic ends is absorbed by astrocytes and metabolized into Gln, which can be absorbed and utilized by the glutamine transporters on glioma cells before being transported to neurons (Strickland and Stoll, 2017).	Provide a special source of amide nitrogen for glioma cells to synthesize purines and pyrimidines.
Special genetic mutations	Mutations or expression changes of IDH, p53, PDGFRA, EGFR, NF1 and other characteristic genes of glioma not only promote glucose uptake and glycolysis in glioma cells, but also induce the infiltration and M2 polarization of GAMs (Wang Q. et al., 2017).	Make glioma cells use the glucose-rich condition of brain TME more efficiently, and promote immune escape.
Participation of microglia	As a special member of glioma TME, microglia are recruited to lesions through CX3CL1/CX3CR1 chemokine channel (Hambardzumyan et al., 2016), while peripheral monocyte-macrophages through the MCP-1/CCR2 channel (Vakilian et al., 2017).	There is a certain difference in the immune recruitment between glioma cells and peripheral tumor cells.
Presence of a biological barrier	The chemokines released by glioma cells recruit most of the immune cells in the TME from the periphery after penetrating BBTB, and immune cells also need to penetrate this barrier to enter the lesion.	There is a barrier for glioma cells to recruit immune cells, which does not exist in peripheral tumors.

## Author Contributions

RQ and YZ contributed equally to this work and worked for the first draft of text writing and illustrations. QL participated in the literature search and revised the introduction part of the manuscript. YL and HF provided professional guidance on disease details and treatment status, and edited the manuscript. All of the authors approved the publication of this manuscript.

## Conflict of Interest

The authors declare that the research was conducted in the absence of any commercial or financial relationships that could be construed as a potential conflict of interest.

## Abbreviations

2-HG: 2-hydroxyglutarate
AA: arachidonic acid
ABCA: ATP-binding cassette sub-family A member
BCAA: branched-chain amino acid
BCKA: branched-chain ketoacid
B-FABP: brain fatty acid binding protein
EAAT: excitatory amino acids transporters
ELOVL2: elongation of very long chain fatty acids protein
FASN: fatty acid synthase
FPR: formylpeptide receptor 1
HK: hexokinase
HMGB: high mobility group protein B
MCT: monocarboxylate transporter
PFK: 6-phosphofructokinase
PGES: prostaglandin E synthase
PKM: pyruvate kinase M
R5P: ribose-5-phosphate
SHMT: serine hydroxymethyltransferase
SOAT: sterol *O*-acyltransferase
SREBP: sterol regulatory element-binding protein
α-KG: α-ketoglutarate.
